# An Automated HPLC Method for SARA Fractionation of Asphalts: Operational Assessment and Comparison with ASTM D4124

**DOI:** 10.1021/acsomega.6c00289

**Published:** 2026-04-22

**Authors:** Giovanni Polacco, Sara Filippi, Chiara Riccardi, Pietro Leandri, Massimo Losa

**Affiliations:** Department of Civil and Industrial Engineering, 9310 University of Pisa, Largo Lucio Lazzarino, 1, 56122 Pisa, Italy

## Abstract

The composition of asphalts and other heavy petroleum-derived materials is usually subdivided into four fractions called saturates, aromatics, resins, and asphaltenes (SARA), which have different polarities and solubilities. The separation and quantification of these fractions using chromatographic methods provide essential information on the colloidal stability, chemical reactivity, and processing behavior. However, the measured distribution of SARA fractions is inherently dependent on the analytical procedure adopted. Based on a liquid chromatographic separation in open columns, ASTM D4124 is still the most widely applied standard, despite being time-consuming, solvent-intensive, and operator-dependent. Although numerous HPLC-based alternatives have been proposed, their broader implementation has been limited by the methodological variability and the lack of a clear framework for interpreting the results across different separation schemes, particularly for asphaltic systems rich in aromatics, resins and asphaltenes. This work presents an automated HPLC workflow for SARA fractionation which is applied to four different penetration-grade asphalts and systematically compared to ASTM D4124. The study highlights the practical aspects of HPLC implementation, including the critical methodological steps that affect the reproducibility and transferability between laboratories. Additional analyses of ASTM-isolated fractions and selected model compounds provide insights into the origins of systematic differences between HPLC- and ASTM-derived SARA compositions. These differences are shown to arise from distinct separation mechanisms and operational cut points rather than from analytical inaccuracies. This reflects the intrinsically method-dependent nature of SARA classification in materials characterized by a continuous distribution of molecular polarity. By clarifying the methodological and conceptual basis of these differences, this work provides practical guidance for the interpretation of HPLC-SARA data and supports more consistent and transferable approaches to asphalt characterization.

## Graphical abstract

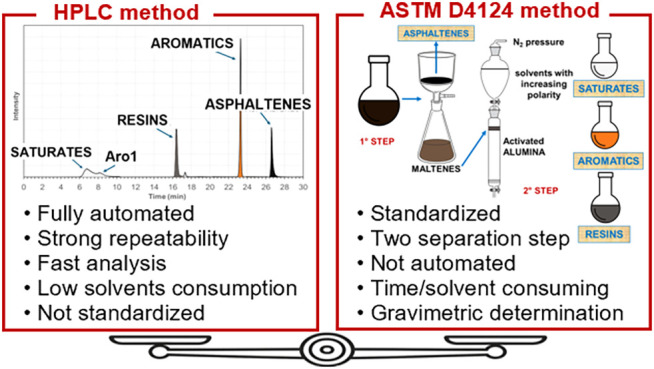

## Introduction

The composition of asphalts and other heavy petroleum-derived materials is commonly divided into four fractions referred to as saturates, aromatics, resins, and asphaltenes (SARA), based on differences in polarity and solubility. The relative abundance of these fractions provides valuable information on colloidal stability, chemical reactivity, compatibility, and processing behavior, and this classification is routinely used in refinery operations, heavy oil upgrading, and asphalt characterization.^1–3^ Different analytical approaches can be used to separate asphalts into these fractions. Most approaches derive conceptually from the procedure originally proposed by Corbett,^4^ based on chromatographic columns and solvents of increasing polarity. The ASTM D4124 standard (Standard Test Methods for Separation of Asphalt into Four Fractions) is the most widely applied reference method. In this protocol, the gravimetric determination of the SARA fractions begins with the preliminary separation of maltenes and asphaltenes. After dissolving the lighter components into *n*-heptane (nC7), the asphaltenes are isolated by filtration, following the methods described in ASTM D6560 and ASTM D3279. This initial step is already time-consuming and requires significant volumes of solvent, which should ultimately be disposed of as waste. The maltene fraction then undergoes a second separation step to obtain the other three subfractions. This is performed using liquid chromatography on activated alumina, which typically involves large column dimensions and a low degree of automation. As a result, this part of the procedure is particularly lengthy and entails substantial solvent consumption. Finally, to gravimetrically quantify the isolated fractions, the solvent must be completely removed from each subfraction, adding further labor-intensive steps to the protocol. Although demanding in terms of time, solvent consumption, and operator effort, ASTM D4124 provides quantitative recovery of isolated fractions which can be further analyzed individually. When only a relative distribution of the four fractions is required, alternative approaches may be considered. Fourier transform infrared spectroscopy (FTIR) has been explored as a rapid indirect method. Weigel and Stephan reported correlations between FTIR and various physical and chemical parameters.^5^ Among the chemical parameters, they included the SARA fractions and concluded that FTIR spectra can be used to describe the asphaltenes and maltenes contents, but not the maltene subfractions, due to their “high structural similarity”. In contrast, Li et al. developed a model to evaluate the content of all the SARA fractions directly from infrared spectra and calibrated the method with data from thin layer chromatography-flame ionization detection (TLC-FID).^6^ Among the chromatographic alternatives, TLC-FID and high-performance liquid chromatography (HPLC) enable limited solvent consumption and a higher degree of automation compared with the open-column method. TLC-FID commercialized under the Iatroscan system, and recognized by the Institute of Petroleum with the standard IP 469/01 (Determination of saturated, aromatic and polar compounds in petroleum products by thin layer chromatography and flame ionization detection) has been used by several research groups.^7–12^ However, sample preparation for TLC-FID is time-consuming, requires multiple solvents and constant operator attention to ensure acceptable repeatability. Moreover, many concerns have been raised within both academia and the road construction industry, since the response of the FID detector depends on the type of components and has poor repeatability. In particular, the flame ionization detector response tends to underestimate resins and asphaltenes due to their lower flammability, while highly polar or strongly adsorbed species may remain near the feed spot and escape detection.^13–15^

HPLC-based SARA methods can be implemented using commercially available columns, valves, and detectors. However, no universally standardized configuration exists, and since the 1970s numerous research groups have proposed specific setups that differ in terms of stationary phases, column arrangements, mobile phases, and operating conditions.^14,16–46^ Despite substantial differences in experimental setups and in the number and type of columns, stationary phases, and mobile phases used, the proposed methods nonetheless share several common features: (1) the first mobile phase is an alkane and saturates are the first components to reach the detectors, as they are not retained in any column; (2) aromatics can either exhibit a longer retention time than saturates, or be adsorbed on a column and then be selectively eluted with other solvents; (3) resins have the strongest interaction with the stationary phase and remain in the very first column from which they are usually released in backflush mode.

The most recent studies also converge on two important aspects. First, the use of an evaporative light scattering detector (ELS) improves quantitative reliability compared to other detectors such as ultraviolet–visible (UV–vis) spectroscopy and the refractive index. Second, the time-consuming preseparation of asphaltenes can be avoided by dissolving the asphalt sample in a polar solvent and injecting it into the HPLC system, where the mixing with the alkane mobile phase induces their precipitation. This has the dual advantage of shortening the overall procedure and almost completely automating the method. Recent representative configurations include the multicolumn systems described by Karevan et al.,^40,41^ and Kheirollahi et al.,^42^ which use three columns in series: poly­(tetrafluoroethylene) (PTFE), cyano and silica. The first column, which acts as a mechanical filter, blocks the asphaltenes, while through an adsorption mechanism the second and third columns retain resins and aromatics, respectively. The three columns are then washed separately with toluene, to solubilize and remove the precipitated asphaltenes and to desorb resins and aromatics. Boysen et al.^43^ used four columns in series: PTFE, glass beads, aminopropyl-functionalized silica and activated silica. Asphaltenes are recovered from PTFE, and resins are recovered by backflushing the aminopropyl column together with the glass beads column. Lastly, aromatics are recovered from the silica column (a progressive increase in the polarity of the solvents enables both asphaltenes and aromatics to be divided into three distinct subfractions). In a very recent work, Atwah et al. simply filtered out the asphaltenes through a silica guard column placed before two cyano and silica columns.^46^ Saturates and aromatics were eluted with *n*-pentane (nC5) in normal flow involving all the columns and then the three columns were backflushed with nC5/dichloromethane (DCM) and isopropyl alcohol/DCM/methanol (MeOH) to elute the resins and asphaltenes, respectively.

While differing in architectural details and analysis times, these approaches illustrate the flexibility of HPLC-SARA and the lack of a single standardized separation logic.

It is worth highlighting that differences in stationary and mobile phases can lead to systematic differences in the reported SARA distributions obtained by chromatographic methods. The same is valid for the operating conditions (such as temperature, pressure, use of solvent gradients) and the choice of operational cut points. This is a direct consequence of the very complex composition of asphalt, where the behavior of many molecules may be borderline between two fractions. Changes in the above-mentioned conditions may shift a molecule from one fraction to another. In other words, their intrinsically overlapping compositional distributions lead to systematic differences in the quantification of SARA fractions when comparing different separation methods.

The objective of the present work is thus to critically evaluate and implement an automated HPLC workflow for SARA determination in asphaltic materials, with particular focus on methodological robustness, solvent management, and practical transferability. The method is applied to several penetration-grade binders and systematically compared with ASTM D4124. In addition to the analytical performance metrics, this study aims to clarify the origins of the systematic differences between HPLC- and ASTM-derived SARA compositions. By analyzing the ASTM-isolated fractions and selected model compounds, the role of the separation mechanisms and operational cut points is examined in detail. By providing practical guidance for laboratory implementation and a conceptual framework for interpreting cross-method differences, this work supports a more consistent and informed use of HPLC-SARA in asphalt characterization.

## Materials and Methods

### Materials

All solvents (nC7, toluene, DCM, MeOH) were HPLC grade, purchased by Merck Life Science and used as received. Alumina (Clearnet Alumina N, AL1000N, 100–200 mesh from Agela Technologies) was activated in a muffle furnace at 425 °C for 16 h. PTFE in the form of powder with an average size of 675 μm was purchased from Merck Life Science and used as received. Hexacosane, 5-α-cholestane, 1-phenyldodecane, pyrene, benzo­(a)­pyrene, 9-anthracenemethanol, 1,10-phenanthroline and acenaphthenequinone were also purchased from Merck Life Science and used as received.

### Asphalts

Four penetration-grade asphalt binders were selected to represent a range of conventional paving grades commonly used in road construction. Table 1 summarizes their main physical properties, including penetration at 25 °C, softening point (ring-and-ball), and performance grade (PG) classification. The selected binders include different consistency levels and thermal performance classes, thus enabling the HPLC-SARA method to be evaluated across materials with different colloidal structures and compositional balance. Penetration values were determined according to ASTM D5, the softening point according to ASTM D36, and the PG classification according to AASHTO M320. These parameters provide a conventional framework for comparing binder stiffness and temperature susceptibility, thus facilitating the interpretation of possible relationships between the SARA distribution and macroscopic performance properties.

**Table 1:** Physical Properties of the Investigated Asphalt Binders: Penetration (25 °C), Softening Point (Ring and Ball), and PG Classification

asphalt	penetration at 25 °C [dmm]	softening point [°C]	PG
35–50	45	53.1	70–22
50–70	65	49.6	58–16
70–100	89	45.2	58–16
160–220	172	35.3	46–28

### SARA Fractions by LC (ASTM D4124)

SARA fractions were determined following a procedure based on the above-mentioned ASTM D4124 standard. After removing insoluble asphaltenes, by treating the asphalt sample (approximately 2.0 g) with nC7 (150 mL) at 40 °C, under constant stirring for 2 h, the maltenes were separated into saturates, aromatics and resins using a glass column (70 cm long and 1.5 cm internal diameter) filled with activated alumina. The stationary phase was accurately prewetted by flowing nC7. A sample of weighed maltenes (approximately 1 g) was dissolved in 10 mL of nC7 and fed to the top of the column. The saturates were then eluted with nC7 (150 mL), followed by toluene (33 mL); aromatics were eluted with toluene (67 mL), followed by a solution of MeOH/toluene 50/50 v/v (75 mL), and resins were eluted with DCM (150 mL). The collected solutions were dried under vacuum to remove the solvents, and the three fractions were weighed separately. Two deviations from the ASTM procedure were introduced. First, instead of using a pump to circulate the solvents from the bottom to the top of the column, the solvent flowed by gravity in the opposite direction with the help of a small overpressure of nitrogen. Second, the asphaltene removal was carried out at 40 °C rather than at the boiling temperature of nC7, thus matching the operating temperature of the HPLC oven. This adjustment ensured comparable asphaltene precipitation conditions in the ASTM and HPLC procedures.

### SARA Fractions by HPLC

The equipment used was a Jasco apparatus with the following components: a Jasco AS4050 autosampler, a Jasco BS-4000–1 bottle stand, a Jasco PU-4180 quaternary gradient pump, five 6-position Jasco HV-4380 switch valves, a Jasco CO-4065 column oven, a Jasco UV-4070 UV–vis detector and a SEDEX 85 LT-ELS detector from SEDERE. The ELS detector was set at 35 °C and 3.3 bar nitrogen flow. Three columns were used, thermostatically controlled at 40 °C: a 50 mm × 7.8 mm stainless steel column filled with PTFE, which acted as a mechanical filter, and two chromatographic columns—a cyano-bonded silica gel (HyperClone 5 μm CN 120 Å, 250 mm × 4.6 mm), and an aminopropyl bonded silica gel (Luna 5 μm Silica 100 Å, 250 mm × 4.6 mm) both provided by Phenomenex, and hereinafter referred to as cyano and amine, respectively.

Approximately 1 g of asphalt was dissolved in 20 mL of DCM at room temperature. The solution was gently stirred with a spatula for a few minutes and left overnight to ensure complete dissolution of all asphalt components. A small quantity of the solution was further diluted in DCM to obtain a concentration of 4 mg/mL, then 10 μL of the solution was injected into the system under an nC7 flow of 1.0 mL/min. The procedure was fully automated by programmed switching of the valves, mobile phase and wavelength of the UV–vis detector. The valves/columns scheme is reported in Figure 1, while Table 2 summarizes the switching times and UV–vis wavelengths. In step 1, with nC7 as the mobile phase, the sample is injected and flows sequentially through the PTFE, cyano and amine columns in series. During this step, asphaltenes precipitate and are retained in the PTFE column. The precipitation of asphaltenes depends on the ability of nC7 to sufficiently reduce the polarity of the dichloromethane solution. However, given the very high nC7/DCM ratio, it is reasonable to assume that the nonsolvent induces complete precipitation. After asphaltenes removal, resins are adsorbed on the cyano column; aromatics are retained on the amine column and only saturates reach the detector. In step 2, the mobile phase is switched to a DCM/MeOH 98/2 solution using a 3 min step gradient, which elutes the resins in backflush mode from the cyano column. In step 3, the DCM/MeOH 98/2 mixture elutes the aromatics from the amine column in forward mode, and in step 4 it elutes the asphaltenes from the PTFE column. Finally, in steps 5–7, a 16 min conditioning step with nC7 is performed to prepare the columns before the next sample. The duration of all the steps was determined through a systematic optimization procedure. Each column was first tested individually to determine its behavior, and the retention time was defined as the point at which the detector’s response ensured that no signal appeared in the following elution steps. Analogously, the duration of steps 5 to 7 guarantees a complete restoration of nC7 in all columns and lines. Overall, the method requires 29 min of data acquisition plus another 16 min for column regeneration, for a total of 45 min per run. Since the flow rate is constantly maintained at 1 mL/min, the total solvent consumption is 45 mL. The recorded wavelength of the UV–vis detector was set at 254, 350, or 500 nm depending on the eluted fraction. The commonly used wavelength is 254 nm for single-ring aromatics, while the optimal wavelength shifts toward the visible range as the number of condensed rings increases.^47^ The chosen wavelengths were not the result of an optimization study since the UV–vis spectrum was not used for quantitative analysis, but rather to detect the presence of aromatic compounds. In addition, the UV–vis detector proved very useful during the process setup, when a huge amount of experimental data was collected to evaluate and optimize the duration of each of the steps described above. Quantitative analysis was thus not necessary as simply detecting the presence of an eluate was sufficient. Therefore, except for the saturates, collecting UV–vis spectra made it possible to avoid the use of ELS, which involves significant nitrogen consumption.

**Figure 1:**
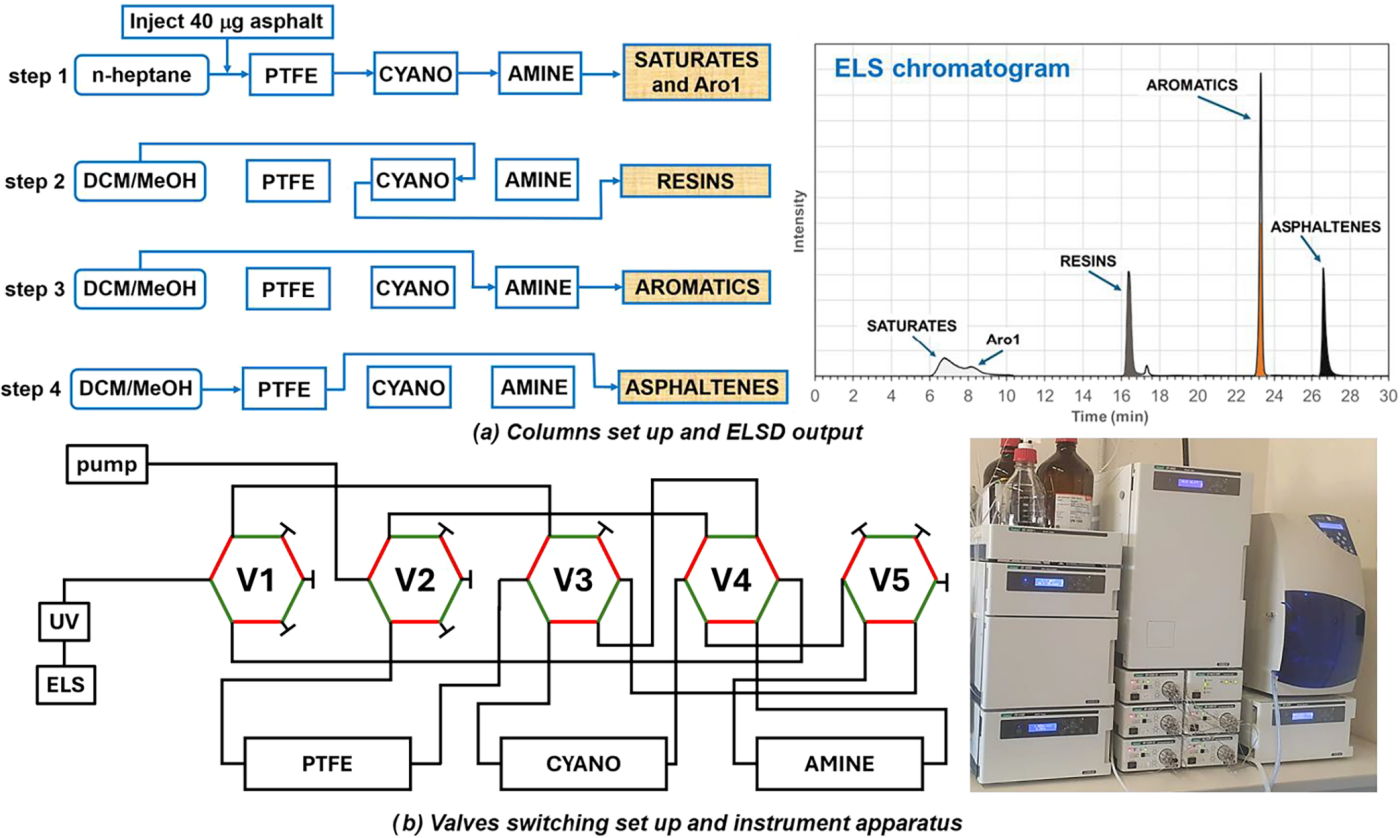
(a) Columns setup and ELS output; (b) valves switching setup and instrument apparatus. V1, V2, V3, V4, V5 = switching valves (green = on; red = off), PTFE = mechanical filter, cyano and amine = HPLC columns, UV–vis and ELS = detectors.

**Table 2:** Setup Switch Valves, Eluents and UV–Vis Wavelengths^a^

step	mobile phase	V1	V2	V3	V4	V5	time (min)	UV–vis (nm)
1	nC7	on	on	on	on	on	0–10	254
2	DCM/MeOH 98/2 v/v	off	on	off			10–20	350
3	DCM/MeOH 98/2 v/v	on	off	off	off		20–26	254
4	DCM/MeOH 98/2 v/v	on	off	on	on	off	26–29	500
5	nC7	on	off	on	on	off	29–32	
6	nC7	on	off	on	on	off	32–41	
7	nC7	on	on	on	on	on	41–45	

a Valve V4 is not involved in step 2, while valve V5 is not involved in steps 2 and 3.

## Results and Discussion

### Development and Optimization of the Automated HPLC Method

When the DCM solution of asphalt is introduced into the HPLC, the presence of nC7 as the mobile phase leads to the precipitation of asphaltenes. The PTFE column then retains the precipitated asphaltenes as a solid phase which is later dissolved by the DCM/MeOH solution. PTFE is expected to enable all other components to pass through and is preferred over other materials due to its physical/chemical inertness. Our experience confirms this hypothesis, but it is worth noting that the PTFE columns created considerable problems when setting up the method. Since commercially available columns packed with PTFE are not available, this column should be prepared in the laboratory. Boysen and Schabron report preparing a 250 mm × 7.0 mm column packed with PTFE particles (250–400 μm), obtained by grinding PTFE boiling stones (D1069193 from Saint-Gobain).^43^ In our study, a PTFE powder, provided by Merck, with a mean particle size of 675 μm, was used to fill a conventional 50 mm × 7.8 mm stainless steel column. However, this configuration immediately led to an overpressure alarm from the equipment, and the powder content in the column had to be significantly reduced (i.e., by adding glass beads). Although this adjustment solved the overpressure problem, it led to a prolonged series of problems related to inconsistent results. Unexpectedly, the PTFE column captured, at least partially, all the SARA fractions. Several samples (including model-molecules and single SARA fractions obtained by the ASTM method) eluted mainly after washing the PTFE column and were therefore detected as asphaltenes. After reducing the dimensions of the PTFE filling to only 10 mm × 7.8 mm, the PTFE column captured all asphaltenes while enabling the other molecules to pass. A second type of PTFE was also tested, which was obtained by grinding a PTFE sheet supplied by Fluorten and selecting particles sized between 250 and 400 μm. However, this material led to similar problems, including overpressure and the undesired retention of all SARA fractions. To the best of our knowledge, such behavior has not been reported by other research groups, which suggests that these issues may be specific to the PTFE powder used in this study. Since PTFE columns are not commercially available, using a manually packed column could raise concerns regarding the variability and transferability of results. However, it is important to consider that the entire ASTM procedure relies on manually packed columns. In addition, in our case, the PTFE powder is commercially available, and the column dimensions are described so that any laboratory could easily reproduce it. Furthermore, since the PTFE column acts as a mechanical filter and not as a chromatographic column, it can also be replaced with alternative commercial columns as in the work of Atwah et al.^46^

Another difficulty in setting up the method was the numerous peaks in both the UV–vis and ELS baselines. These peaks, associated with valve switching and solvent changes, exhibited significant magnitude and often overlapped with analytical signals, thus making the baseline subtraction challenging and the quantitative analysis nearly impossible. Similar problems with the baseline were described by Karevan et al., who completely removed the nC5 (first mobile phase) before introducing toluene, as the eluent for all the columns. The nC5 was removed with the help of propane and vacuum^40^ or ethane and vacuum.^41^ Although effective, this solution is demanding in terms of equipment and gas handling. Moreover, the sweeping of nC5 and subsequent conditioning of the columns with toluene require extra time which in the end leads to a cycle of 6 h for each single run. Two main factors contribute to the noisy baseline. First, valve switching and changes in the mobile phase disturb the detectors (especially the UV–vis). A single eluent was used and the whole procedure therefore required only a solvent change (from nC7 to the DCM/MeOH solution). Performing the solvent change with a step gradient further reduces the undesired noise. Second, the equipment includes five valves and a significant number of connections that are filled with solvent. Due to changes in the valves position, some of the connecting lines remain isolated. The solvent trapped in these isolated lines can re-enter the flow path upon subsequent valve switching. If, in the meantime, the main solvent has been changed, small plugs from the previous solvent are introduced into the system, thus generating disturbances that may affect not only the baseline but also the output. This explains the conditioning steps (steps 5–7 described in Table 2), during which nC7 is flushed through the system to ensure the complete removal of the DCM/MeOH solution from all the columns and lines.

Figure 2 compares the UV–vis and ELS baselines obtained by injecting 10 μL of pure DCM, which was the solvent used for the preparation of asphalt solutions. The UV–vis baseline is slightly shifted upward along the *y*-axis to avoid overlap; however, neither curve has been modified or normalized. Compared with the ELS trace, the UV–vis baseline has two additional peaks that correspond to the valve switches around 20 and 26 min. These two peaks have a comparable or even higher intensity than those of the samples, making them difficult to subtract without introducing errors or artifacts. However, the baseline is smooth and clean in the first 10 min of elution, which is the interval in which the UV–vis was used to interpret the chromatograms, as described below. In contrast, the ELS detector produces a very smooth baseline that can be easily handled. A small peak around 17.3 min, in the resins region (step 2), initially raised some concern. When a sample containing resins is loaded in the system, the main peak corresponding to their elution ends at approximately 17 min and is followed by a small one at 17.3 min, which is notably more intense than the corresponding peak in the baseline. After several tests, this peak was excluded from the integration of the resins fraction since its amplitude was shown to be independent of both the concentration of the sample and the volume of solution injected into the HPLC. Figure 3 shows the chromatograms obtained in the resins region while feeding solutions with different concentrations of asphalt 50–70. Interestingly, a similar peak was observed in the chromatogram reported by Adams et al.^44^

**Figure 2:**
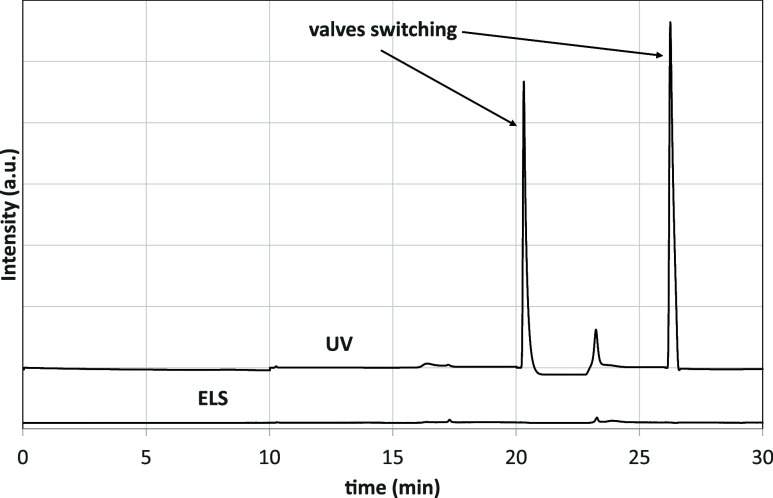
Chromatograms recorded by UV–vis and ELS detectors, when injecting 10 μL of pure DCM without dissolving any asphalt sample. The signals due to the valve switching (visible only in the UV–vis chromatogram) are indicated by the arrows.

**Figure 3:**
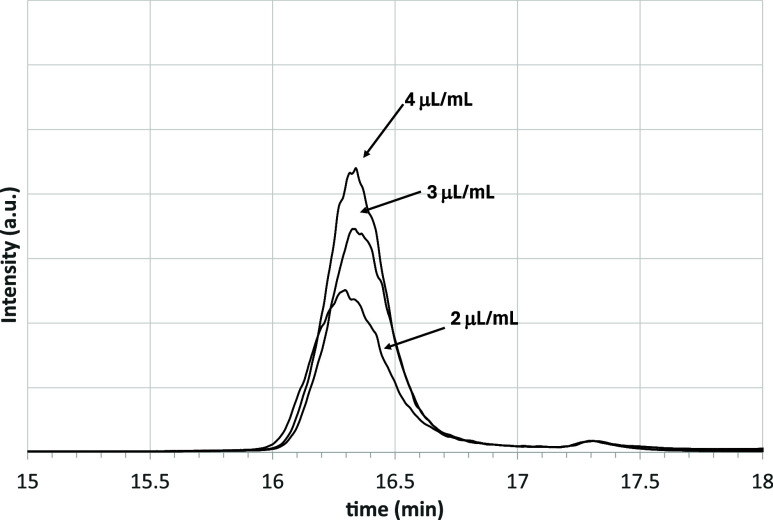
Chromatogram in the resins region analyzing solutions with three different concentrations of asphalt 50–70.

Before analyzing a new set of samples, a preliminary test was always performed by injecting 10 μL of pure DCM into the system. The corresponding chromatogram produced by the ELS detector was used as the baseline, which was simply subtracted mathematically from subsequent chromatograms.

### Identification of the SARA Fractions

Figure 4 shows an example of a chromatogram obtained for asphalt 50–70 using both UV–vis and ELS detectors.

**Figure 4:**
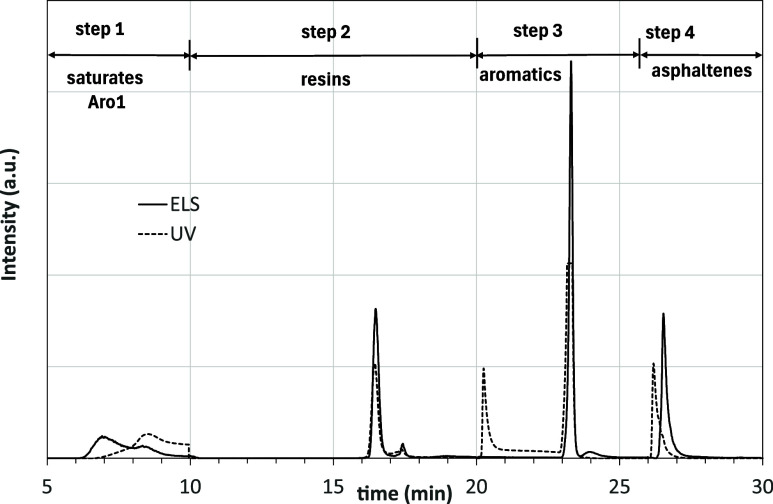
ELS and UV–vis chromatograms for an asphalt 50–70 sample.

Based on the programmed switches of the valves, the signals in Figure 4 can be easily attributed to the four SARA fractions, except for saturates. In the time interval between 6 and 10 min, the ELS shows two peaks. If these peaks were only due to saturates, then the UV–vis spectra would be flat. In contrast, the UV–vis detects aromatic rings in the second peak. In Figure 4, the absorbance at a single wavelength is recorded as a function of time; however, the UV–vis detector can also obtain a whole spectrum at specific times. Figure 5 shows the UV–vis spectra recorded after 6.5 and 7.7 min, which correspond to the first and second peaks in the ELS signal.

**Figure 5:**
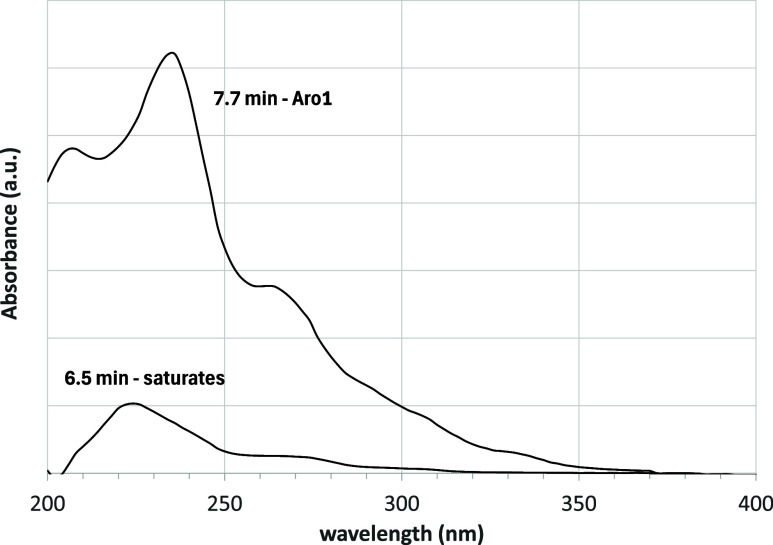
UV–vis spectra recorded at the retention time corresponding to the maximum of the saturates and Aro1 peaks, respectively, for the asphalt 50–70 sample shown in Figure 4.

Only the eluates of the second peak are clearly visible in the UV–vis signal. Therefore, after the first saturates, some molecules with a mixed paraffinic (or naphthenic) and moderate aromatic character appear. This difficulty in separating saturates from aromatics was already described by Srinivasan et al., who highlighted how the degree of separation depends on the choice of stationary and mobile phases.^48^ Moreover, a similar behavior was described by Adams et al.^45^ who named these compounds “Aro1” based on the assumption that they have a single aromatic ring. The same nomenclature is used here.

Since Aro1 belongs to the aromatics fraction, their contribution should be subtracted to evaluate the real content of saturates in the ELS area between 6 and 10 min. A deconvolution into Gaussian curves (Figure 6) indicates that three of them are necessary (and sufficient) to accurately describe the curve. From left to right, the first two peaks are due to paraffinic and naphthenic saturates, respectively, and the third one to Aro1 compounds.^45^

**Figure 6:**
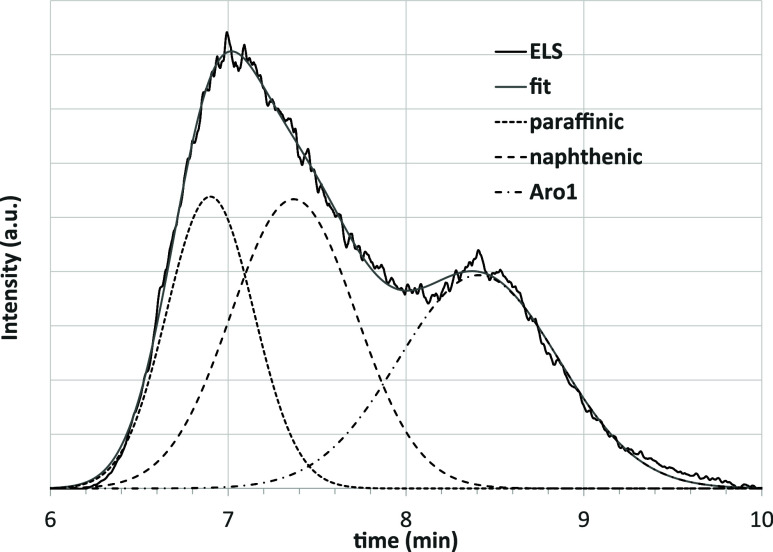
Deconvolution into three Gaussian curves of the ELS signal recorded in the 6–10 min time interval for the sample shown in Figure 4.

Considering this deconvolution and attributing the area corresponding to Aro1 to the aromatic fraction, the correct percentage of the four SARA fractions can be calculated. For example, the saturates and aromatic fractions of asphalt 50–70 without deconvolution would be 22.3 and 32.2%, respectively, while they become 13.9 and 41.2 after considering the area assigned to Aro1 compounds. Deconvolutions for all the chromatograms were performed using Origin software, and the coefficient of determination *R* ^2^ was always greater than 0.995. The deconvolution does not impose an artificial chemical distinction but provides a quantitative allocation of partially overlapping chromatographic contributions, which is supported by independent UV–vis spectral evidence collected in the same elution interval. The deconvolution to separate the contributions of saturates and aromatics is justified by the fact that the boundary between the two fractions is well-defined from a chemical perspective. The saturates are paraffinic-naphthenic molecules, without aromatic rings. Aromatics may have paraffinic-naphthenic branches, but they contain at least one benzene ring. However, although there are no ambiguities from a chemical perspective, aromatics with a single ring and high molecular-weight saturated branches may have a similar polarity to saturates. This is why some Aro1 compounds overlap with saturates in the chromatogram. This overlapping denotes a similar chromatographic behavior but corresponds to a cross-contamination from a chemical perspective. This cross-contamination between saturates and light aromatics may become particularly significant when considering a potential application to crude oils or light petroleum fluids. In the case of asphaltic materials, however, the relative proportion of light aromatics and low-molecular-weight saturates is comparatively limited, and the integrated area assigned to each fraction was found to be highly repeatable. In addition, the systematic comparison with ASTM D4124 shows consistent trends across binders, which suggests that the observed overlap does not introduce uncontrolled variability in the context of asphalt characterization. However, we acknowledge that fraction purity in this elution region cannot be rigorously guaranteed under the current configuration. A dedicated study aimed at optimizing column and solvent combinations to improve the resolution of light aromatics is currently ongoing, and further validation is required before extending the method to lighter petroleum systems.

It is worth underlining that, unlike saturates and light aromatics, a clear chemical differentiation between aromatics and resin, and between resins and asphaltenes is not possible. Therefore, the boundaries among these three fractions are determined by the separation method and the very concept of cross-contamination becomes insignificant.

### Percentage Uncertainty and Repeatability

Once the method had been set up, a high number of repeatability tests were performed. The chromatograms obtained while repeating the analysis on the same sample were almost completely superimposed. Figure 7 shows five chromatograms of asphalt 50–70. These are raw data; the chromatograms were then normalized and slightly shifted vertically to prevent the overlap. To quantify the SARA fractions, the peaks were integrated using only ELS data.

**Figure 7:**
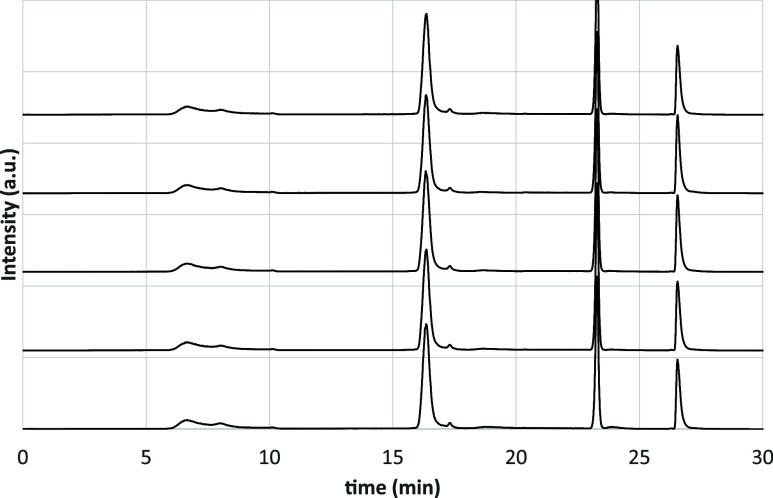
Chromatograms of five different samples of asphalt 50–70 prepared using the same procedure.

Once the configuration had been selected, the repeatability of the results was quantitatively evaluated by comparing 5 samples for each asphalt. The results are reported in Tables 3a, 3b, 3c, and 3d. The half-range (Δ/2) and percentage uncertainty (%*U*) were evaluated as

$$\Delta /2=\frac{\left(A_{\max}-A_{\min}\right)}{2}$$

$$\%U=\frac{\Delta /2}{A_{\mathrm{av}}}\times 100$$

where *A*_max_ and *A*_min_ are the maximum and minimum values of the measured content for each SARA fraction and *A*_av_ is the average value. The 95% repeatability limit (*R*_95_) was evaluated as *R*_95_ = 2.77σ, where σ is the standard deviation for each set of data.

**Table 3a:** Asphalt 35–50

sample N°	saturates	aromatics	resins	asphaltenes
1	17.9	29.6	28.6	23.9
2	17.3	28.5	29.2	25.0
3	17.6	29.8	29.5	23.1
4	17.6	28.4	29.1	24.9
5	18.1	29.6	28.8	23.5
Δ/2	0.40	0.70	0.45	0.95
%*U*	2.3	2.3	1.5	3.9
*R*_95_	0.781	1.630	0.869	2.069

**Table 3b:** Asphalt 50–70

sample N°	saturates	aromatics	resins	asphaltenes
1	14.6	40.4	24.1	20.9
2	14.0	40.7	25.4	19.9
3	14.3	39.8	24.9	21.0
4	15.3	40.2	25.1	19.4
5	14.1	39.6	25.8	20.5
Δ/2	0.65	0.55	0.85	0.80
%*U*	4.4	1.4	3.4	3.9
*R*_95_	1.28	1.11	1.56	1.67

**Table 3c:** Asphalt 70–100

sample N°	saturates	aromatics	resins	asphaltenes
1	14.8	42.4	23.6	19.2
2	16.3	41.1	24.2	18.4
3	16.2	41.8	23.6	18.4
4	15.3	41.5	23.4	19.8
5	15.6	40.9	24.5	19.0
Δ/2	0.75	0.75	0.55	0.70
%*U*	4.8	1.8	2.3	3.8
*R*_95_	1.55	1.47	1.17	1.49

**Table 3d:** Asphalt 160–220

sample N°	saturates	aromatics	resins	asphaltenes
1	25.3	36.4	22.0	16.3
2	24.8	35.4	21.3	18.5
3	26.0	35.5	22.1	16.4
4	24.7	35.2	21.7	18.4
5	25.4	36.1	21.2	17.3
Δ/2	0.65	0.60	0.45	1.10
%*U*	2.5	1.7	2.0	6.1
*R*_95_	1.26	1.22	0.98	2.53

It should be emphasized that the use of the cyano and amine columns is known to cause irreversible adsorption. In the case of asphalts, some of the highly polar resins (asphaltenes have already been removed) may be involved. This may alter the results and shorten the lifespan of the column. Unfortunately, when dealing with such complex materials as asphalts that contain a huge variety of different chemical compounds, this is an unavoidable problem. However, since these columns are used in most of the literature,^17,20–25,28,29,31,32,34,36,37,39,40,43,46,49,50^ it is generally assumed that the irreversibly adsorbed molecules do not significantly alter the quantitative analysis of a single run. However, for this and other reasons, the columns may deteriorate, and occasionally the need for replacement should be evaluated. For this reason, and for internal use only, asphalt 50–70 was used to verify the integrity of the columns by repeating its analysis. To date, after hundreds of samples, no significant deviations from the values reported in Table 3 have been noted.

Currently, reproducibility data obtained by comparing results from different laboratories are not available since the method has only just been developed and is described here for the first time. However, since all components are commercially available, the detailed description of the equipment components, setup, and analytical procedure enables the method to be replicated in other laboratories. The total setup requires an operator with conventional HPLC skills and has an almost identical cost to a TLC-FID system. As already stated, the only handmade element is the PTFE column, which is filled with commercial PTFE. Due to its role as a physical filter, the PTFE column is not involved in the process of chromatographic separation, which may affect whether a molecule belongs to one fraction rather than another. The PTFE should only stop the solid fraction, and its functionality can be easily tested and validated as previously described. The introduction of a manually packed column should therefore in no way compromise the transferability and reproducibility of the method.

### Comparison with ASTM D4124 and Analysis of Methodological Differences

Table 4 reports the percentages of SARA fractions for the four penetration-grade asphalts, together with values derived from ASTM D4124.

**Table 4:** Percentages of SARA Fractions Determined by HPLC and ASTM D4124

asphalt	method	saturates	aromatics	resins	asphaltenes
35–50	HPLC	17.7	29.2	29.1	24.0
35–50	ASTM	13.2	40.5	18.7	27.6
50–70	HPLC	14.5	40.1	25.0	20.4
50–70	ASTM	13.7	41.1	18.7	26.5
70–100	HPLC	15.6	41.6	23.9	18.9
70–100	ASTM	14.9	42.5	16.5	26.1
160–220	HPLC	25.2	35.8	21.6	17.4
160–220	ASTM	14.4	38.0	25.2	22.4

Systematic differences between the two sets of results can be clearly observed: the HPLC method provides higher saturate and resin contents and lower aromatic and asphaltene fractions compared to ASTM. These discrepancies are consistently reproduced across all samples and derive from the fundamentally different separation mechanisms of the two procedures. This method-dependent fractionation behavior was already described by Srinivasan et al., who showed how hydrocarbon fractionation outcomes are strongly influenced by sorbent selection and separation design.^48^

To better understand the origin of these differences, the individual fractions obtained with the ASTM procedure were analyzed separately with HPLC. In what follows, they are referred to as ASTM/asphalt/name of fraction (i.e., ASTM/50–70/saturates). Starting with the ASTM/saturates, indifferently from the analyzed asphalt, the HPLC chromatogram shows a signal only in the expected time interval (6–10 min) and the peak corresponding to Aro1 compounds is absent (which is confirmed by the UV–vis detector). This indicates that the activated alumina has a higher selectivity between saturates and aromatics than the amine column. The peak of the ASTM-saturates is asymmetric and can be satisfactorily described with a deconvolution into two Gaussian curves, thus confirming the presence of both paraffinic and naphthenic compounds. For example, Figure 8 shows the chromatogram for ASTM/50–70/saturates, in the time interval of interest, together with the deconvolution in two peaks. The chromatogram of the whole asphalt, with the Aro1 compounds, is also added to the graph for comparison.

**Figure 8:**
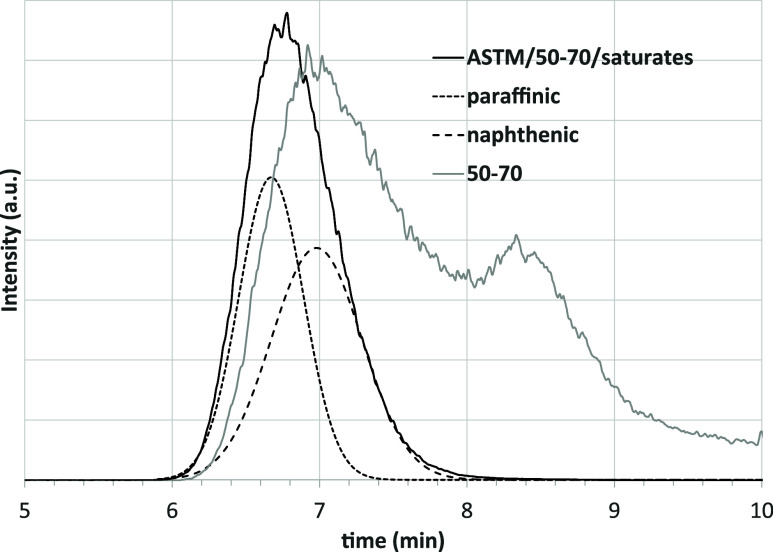
HPLC of asphalt 50–70 and its ASTM-saturates fraction. The curves referred to as “paraffinic” and “naphthenic” correspond to the deconvolution into two Gaussian curves.

The behavior of the ASTM–aromatics, −resins and −asphaltenes fractions was markedly different. When reanalyzed by HPLC, each of these ASTM-derived fractions showed signals in more than one SARA region (Figure 9).

**Figure 9:**
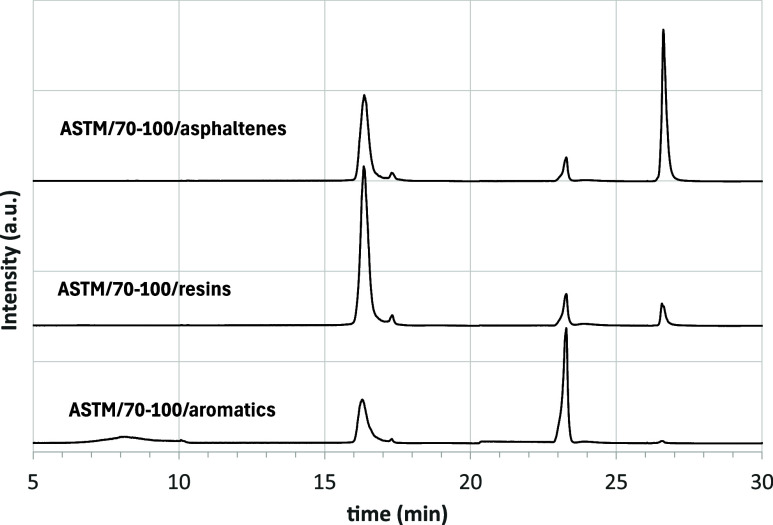
HPLC chromatograms of the ASTM fractions of asphalt 70–100. The three chromatograms are vertically shifted for better readability of the image.

The ASTM aromatics fraction contained a significant number of resins. The ASTM resins fraction exhibited contributions from both aromatics and asphaltenes, and the ASTM asphaltenes fraction showed substantial resin content. These observations reflect the intrinsic difficulty of applying sharp cut points to a material such as asphalt, whose molecular composition spans a continuum of polarities rather than discrete classes. The chromatographic bands formed in the alumina column are broad and partially overlapping, making visual cut-point selection particularly sensitive to operator judgment. To better understand these chromatograms, a recap of the ASTM procedure is helpful. The first step is the separation of asphaltenes, as a brown-black powder, from maltenes that appear brown and very viscous. Maltenes are then dissolved in nC7 and fed to the column (Figure 10).

**Figure 10:**
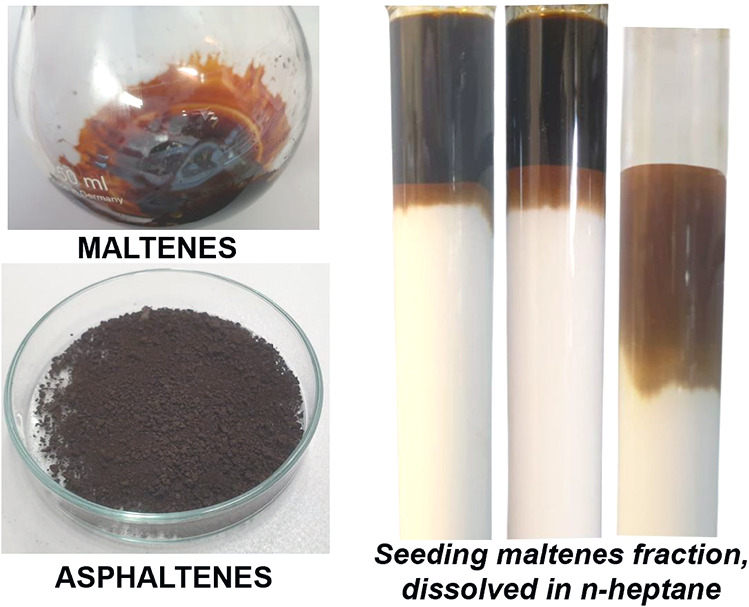
Asphaltenes and maltenes and seeding of the alumina column.

During the first elution with nC7, saturates migrate rapidly, while aromatics accumulate as a yellow band and resins remain near the seeding point. The transition from nC7 to toluene mobilizes the remaining saturates and initiates the downward movement of the aromatics. The first cut point should therefore be placed before the aromatics reach the column outlet (Figure 11a–c). The second cut point—between aromatics and resins—is even more challenging, as the brown resin front advances gradually and contains numerous molecules with a polarity similar to that of the aromatics (Figure 11d–g). This structural continuity explains why ASTM-derived fractions contain significant proportions of neighboring SARA components. Lastly, DCM elutes the resins (Figure 11h–j), and the column remains a homogeneous light brownish color, thus indicating the presence of a few retained molecules (Figure 11j).

**Figure 11:**
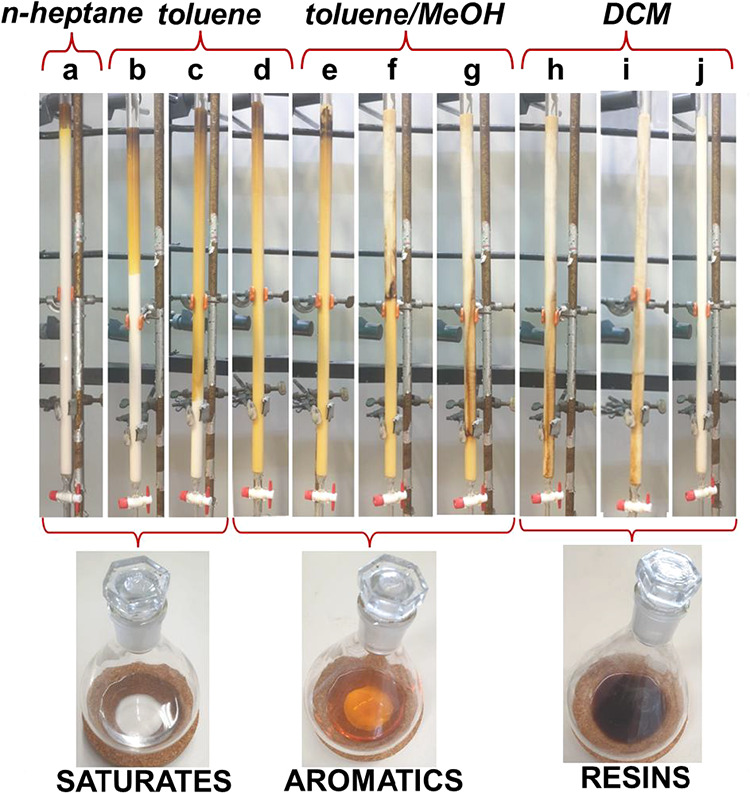
Alumina column during the ASTM procedure and collected fractions.

These observations are fully consistent with the operation of the HPLC system. In the automated method, saturates and Aro1 are not retained and elute in Step 1; resins are selectively retained by the cyano column and elute in Step 2; higher-order aromatics (Aro2–Aro3) are retained by the amine column and elute in Step 3; and asphaltenes, precipitated in situ, are released from the PTFE filter in Step 4. The use of three distinct stationary phases therefore imposes sharper selectivity boundaries than the single alumina column used in ASTM D4124. This leads to a clearer chromatographic assignment of borderline molecules: monocyclic aromatics (Aro1) migrate with the saturates, many aromatic–polar species migrate with the resins, and small asphaltene-like molecules may behave as resins under the operating conditions of the HPLC system.

In fact, small shifts in the ASTM collection boundaries produce noticeable changes in the HPLC profiles of the ASTM fractions, further highlighting the operational nature of the ASTM separation. Figure 12 shows the three ASTM-fractions for asphalt 50–70 when the second cut point is shifted backward in time. ASTM/50–70/aromatics has a single peak, and no resins are detected. At the same time, if the second cut point is brought backward in time, the ASTM-resins fraction shows a higher content of aromatics.

**Figure 12:**
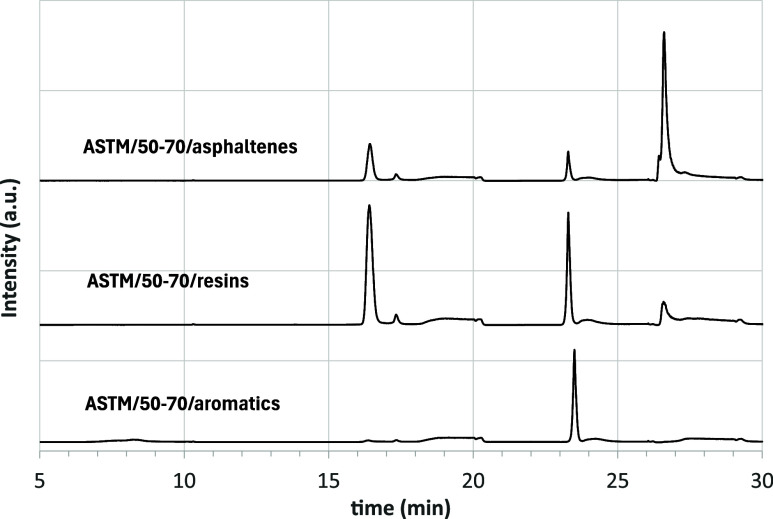
HPLC chromatograms of the ASTM fractions of asphalt 50–70 when the first cut point is brought forward in time (the three chromatograms are vertically shifted for a better readability of the image).

It is worth noting that a correction of the percentages obtained by the ASTM procedure, based on their HPLC chromatograms, does not fill the gap in the compositions measured by the two techniques. This confirms that the chemical structure does not unambiguously identify which SARA fraction a molecule belongs to.

In summary, the systematic differences observed between ASTM D4124 and the automated HPLC procedure arise from the fundamentally different separation mechanisms of the two methods. In the ASTM approach, broad and partially overlapping bands, especially for aromatics and resins, combined with visually determined cut points lead to fractions that appear to be affected by cross-contamination when analyzed by HPLC. In contrast, the HPLC system separates fractions according to well-defined interactions with the cyano and amine stationary phases, which results in a clearer assignment of borderline molecules and higher selectivity. HPLC typically yields higher saturates and resins fractions and lower aromatics and asphaltenes fractions than ASTM. This does not reflect an analytical discrepancy but the intrinsic operational nature of SARA classification in complex materials. It highlights the importance of using a single, well-defined method to compare different asphalts.

### Use of Model Compounds

In the literature cited so far, there are many examples of model molecules used either to calibrate the method^20,21^ or to identify the composition of the different SARA fractions. For example, model compounds were used to subdivide the aromatics based on the number of benzene rings in the molecule.^22,27,31,39,46,49^ Recently, Adams et al. carried out an extensive and very interesting study, in which a huge number of model molecules were analyzed and assigned to the different fractions identified with HPLC.^45^ In this work, the aromatics were subdivided into three subcategories of increasing polarity, named Aro1, Aro2, and Aro3, which can be separated chromatographically. Aro1 consists of molecules with one aromatic ring, while Aro2 and Aro3 mostly contain a higher number of rings. Analogously, other model compounds were used to describe the chemical structure of the other SARA fractions.

Among the model compounds used by Adams et al. the following were used to verify their behavior in our setup: hexacosane (saturates, paraffinic), 5-α-cholestane (saturates, naphthenic), 1-phenyldodecane (Aro1), pyrene (Aro2), benzo­(a)­pyrene (Aro3), 9-anthracenemethanol and 1,10-phenanthroline (resin) and acenaphthenequinone (asphaltenes).

Figure 13 shows the HPLC chromatograms obtained. All molecules attributed to saturates and aromatics produced the expected results. The paraffinic compound showed a slightly lower retention time than the naphthenic compound, which in turn eluted before 1-phenyldodecane, thus confirming the previously described deconvolution analysis. Analogously, pyrene (Aro2) and benzo­(a)­pyrene (Aro3) were eluted from the amine column, as expected. In contrast, some discrepancies in the resins and asphaltenes fractions were observed, since both 9-anthracenemethanol and acenaphthenequinone fall into resins fraction although the latter was selected as representative of asphaltenes. Finally, 1,10-phenanthroline was insoluble in nC7 at 40 °C and behaved as asphaltene.

**Figure 13:**
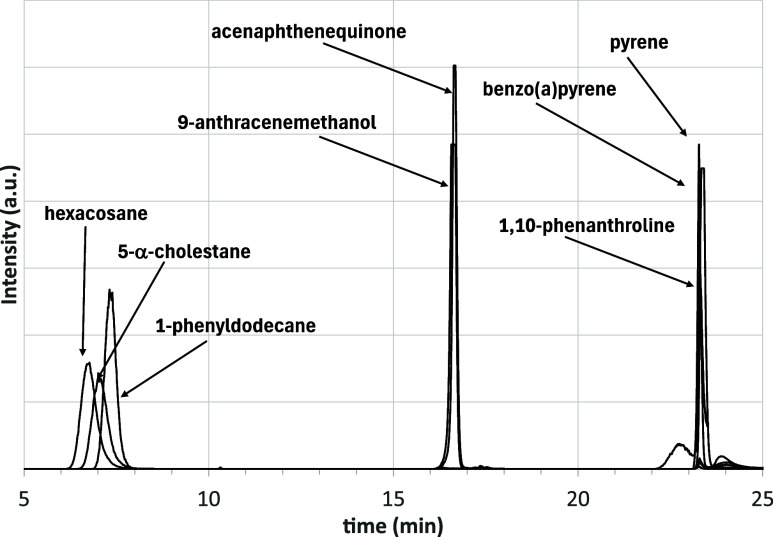
HPLC chromatograms of the model compounds.

These inconsistencies should not be surprising and do not indicate that one of the methods is incorrect. It is a further confirmation that a molecule cannot be unambiguously classified as aromatic, resin or asphaltene, since this assignment depends on both its chemistry and the operating conditions, which include mobile and stationary phases.

## Conclusions

This study presents the development and critical evaluation of an automated HPLC workflow for SARA determination in asphalts. Rather than proposing a fundamentally new chromatographic architecture, the work focuses on practical aspects of method implementation, solvent management, and automation, with specific focus on the transferability to routine laboratory practice. The final setup requires 45 min per analysis, including column reconditioning, with controlled solvent consumption and reproducible fraction quantification.

The systematic comparison with ASTM D4124 confirms that differences between HPLC- and ASTM-derived SARA compositions are inherent to the distinct separation principles of the two methods. The ASTM procedure relies on adsorption in a single alumina column and visually defined collection cut points. On the other hand, the HPLC system uses selective interactions with cyano and amine stationary phases under programmed elution conditions. Consequently, borderline molecules, especially within the aromatic, resin, and asphaltene regions, are assigned differently. These discrepancies do not reflect analytical inaccuracies but arise from method-dependent operational definitions applied to chemically complex materials in which compositional differences evolve gradually rather than forming clearly discrete classes. SARA fractions should therefore be regarded as operationally defined groups rather than chemically discrete entities.

The analysis of ASTM-isolated fractions and selected model compounds further demonstrates that SARA boundaries are governed by separation conditions and cannot be defined based on molecular structure alone. Accordingly, attempting to replicate ASTM results through HPLC, or vice versa, is conceptually inappropriate. Significant compositional comparisons require the consistent use of a single analytical framework.

From an operational perspective, the robustness of the proposed configuration was verified through repeatability testing and dedicated validation procedures. In particular, the manually packed PTFE filtration column used for in-line asphaltene retention proved stable and reliable after optimization of the packing configuration. Since the PTFE element acts exclusively as a mechanical filter and does not participate in chromatographic selectivity, it does not influence the assignment of molecules to specific SARA fractions.

Beyond the analytical setup itself, this work provides practical guidance for laboratories implementing HPLC-SARA in asphalt characterization. It offers a clearer conceptual framework for interpreting differences between HPLC- and ASTM-based SARA determinations. This is essential when SARA data are used for compositional comparison, aging studies, or performance-related correlations in asphalt binders.

Future work will address interlaboratory validation, long-term column stability, and further assessment of fraction purity to support wider methodological harmonization.

## Abbreviations

Ac: acetone
DCM: dichloromethane
ELS: evaporative light scattering
HPLC: high performance liquid chromatography
LC: liquid chromatography
LC/FID: liquid chromatography/flame ionization detector
MeOH: methanol
nC5: *n*-pentane
nC7: *n*-heptane
PTFE: poly­(tetrafluoroethylene)
SARA: saturates aromatics resins asphaltenes
TLC-FID: thin layer chromatography-flame ionization detector
UV–vis: ultraviolet–visible
